# Strengthening facility-based integrated emergency care services for time sensitive emergencies at all levels of healthcare in India: An implementation research study protocol

**DOI:** 10.1186/s12961-024-01183-x

**Published:** 2024-09-09

**Authors:** Tej Prakash Sinha, Sanjeev Bhoi, Dolly Sharma, Sushmita Chauhan, Radhika Magan, Ankit Kumar Sahu, Stuti Bhargava, Patanjali Dev Nayar, Venkatnarayan Kannan, Rakesh Lodha, Garima Kacchawa, Narendra Kumar Arora, Moji Jini, Pramod Kumar Sinha, Satyajeet Verma, Pawan Goyal, K. V. Viswanathan, Kemba Padu, Pallavi Boro, Yogesh Kumar, Pratibha Gupta, Srikanth Damodaran, Nasar Jubair

**Affiliations:** 1 Department of Emergency Medicine, All India Institute of Medical Sciences, New Delhi, India; 2 Division of NCD, ICMR, New Delhi, India; 3 WHO SEARO, New Delhi, India; 4 OSD, Health, NITI Aayog, New Delhi, India; 5 Department of Paediatrics, AIIMS, New Delhi, India; 6 Department of Gynaecology, AIIMS, New Delhi, India; 7 INCLEN, New Delhi, India; 8 Tomo Riba Institute of Health & Medical Sciences, Naharlagun, Arunachal Pradesh India; 9 Medicine Department, Anugrah Narayan Magadh Medical College, Gaya, Bihar India; 10 Rajarshi Dashrath Autonomous State Medical College, Ayodhya, Uttar Pradesh India; 11 Shaheed Hasan Khan Mewati Government Medical College Nalhar Hospital, Nuh, Haryana India; 12 Emergency Department, Trivandrum Medical College, Trivandrum, Kerala India; 13 Department of Emergency & Trauma, Tomo Riba Institute of Health & Medical Sciences, Naharlagun, Arunachal Pradesh India; 14 Department of Community Medicine, Tomo Riba Institute of Health & Medical Sciences, Naharlagun, Arunachal Pradesh India; 15 Department of Opthalmology, Shaheed Hasan Khan Mewati Government Medical College Nalhar Hospital, Nuh, Haryana India; 16 Department of Community Medicine, Rajarshi Dashrath Autonomous State Medical College, Ayodhya, Uttar Pradesh India; 17 Emergency & Trauma Care, Trivandrum District Hospital, Thiruvananthapuram, Kerala India; 18 Department of Emergency Medicine, Anugrah Narayan Magadh Medical College, Gaya, Bihar India

**Keywords:** Emergency care services, India, Implementation research, Time-sensitive conditions

## Abstract

**Background:**

The healthcare system in India is tiered and has primary, secondary and tertiary levels of facilities depending on the complexity and severity of health challenges at these facilities. Evidence suggests that emergency services in the country is fragmented. This study aims to identify the barriers and facilitators of emergency care delivery for patients with time-sensitive conditions, and develop and implement a contextually relevant model, and measure its impact using implementation research outcomes.

**Methods:**

We will study 85 healthcare facilities across five zones of the country and focus on emergency care delivery for 11 time-sensitive conditions. This implementation research will include seven phases: the preparatory phase, formative assessment, co-design of Model “Zero”, co-implementation, model optimization, end-line evaluation and consolidation phase. The “preparatory phase” will involve stakeholder meetings, approval from health authorities and the establishment of a research ecosystem. The “formative assessment” will include quantitative and qualitative evaluations of the existing healthcare facilities and personnel to identify gaps, barriers and facilitators of emergency care services for time-sensitive conditions. On the basis of the results of the formative assessment, context-specific implementation strategies will be developed through meetings with stakeholders, providers and experts. The “co-design of Model ‘Zero’” phase will help develop the initial Model “Zero”, which will be pilot tested on a small scale (co-implementation). In the “model optimization” phase, iterative feedback loops of meetings and testing various strategies will help develop and implement the final context-specific model. End-line evaluation will assess implementation research outcomes such as acceptability, adoption, fidelity and penetration. The consolidation phase will include planning for the sustenance of the interventions.

**Discussion:**

In a country such as India, where resources are scarce, this study will identify the barriers and facilitators to delivering emergency care services for time-sensitive conditions across five varied zones of the country. Stakeholder and provider participation in developing consensus-based implementation strategies, along with iterative cycles of meetings and testing, will help adapt these strategies to local needs. This approach will ensure that the developed models are practical, feasible and tailored to the specific challenges and requirements of each region.

**Supplementary Information:**

The online version contains supplementary material available at 10.1186/s12961-024-01183-x.

## Introduction

An emergency Care System (ECS) is an essential component of universal health coverage and for large population around the world, as it is the first point of contact with the healthcare system [1]. A World Health Organization report in 2020 demonstrated that 10 of the leading causes of death in low- and middle-income countries (LMICs) are related to emergency conditions such as heart disease, stroke, lower respiratory infections, etc. [2]. Despite the higher burden of emergency conditions, emergency services utilization is substantially lower in LMICs, possibly due to limited access to ECS [3]. The 72nd World Health Assembly on 30 May 2019 emphasized that many health interventions are time sensitive and that an integrated ECS provider is an effective solution for the delivery of accessible, quality and time-sensitive healthcare services in the case of acute illnesses and injuries throughout the life-course [4].

India is now the most populous country in the world, with one sixth of the world’s population [5]. However the ECS in the country is inadequately developed, leading to high emergency-related mortality [6]. In 2019, the National Institute for Transforming India (NITI) Aayog and All India Institute of Medical Sciences (AIIMS) New Delhi conducted a pan-India study to assess the status of emergency and injury care at government (tertiary and secondary care) and private hospitals. The study demonstrated that, although emergency and injury cases accounted for one tenth of all patients visiting the health facilities, only 3–5% beds are available for catering to emergency admissions. A dedicated emergency department (ED) was available only in half of the studied facilities, whereas a dedicated triage area was present only in one third of these facilities. Most facilities lacked trained healthcare staff who are dedicated to and trained for ED services and lacked standard operating procedures for emergency care. There were no provisions for dedicated funding, legislation and regulations within the system [7]. This led to the recommendation for “developing a robust integrated ECS system which can comprehensively address all medical and surgical emergencies,inclusive of trauma related care across all levels of healthcare facilities” [6, 7].

Time-sensitive conditions (TSC) are critically important in ECS due to their potential for severe consequences such as high mortality and morbidity. However, the lack of a clear definition for TSCs, as highlighted in a study by Wibring et al., leads to inconsistent treatment approaches and challenges in recognizing and managing these conditions effectively [8]. The Indian Council of Medical Research (ICMR), NITI-AIIMS study, and insights from the various registries of India have identified a high mortality rate associated with these conditions in India. Consequently, the authors of this study have deliberately chosen to focus on specific TSCs including chest pain, stroke, trauma, shock, unconsciousness, respiratory distress, poisoning, snake bite, burns, post-partum haemorrhage and seizures, with a particular emphasis on paediatric emergencies, to address these critical health challenges more effectively.

Despite the existence of strong, research-backed protocols to guide the management of these time-sensitive conditions, the application of these practices to patient care remains suboptimal in developing countries including India [9]. This evidence-practice gap, that is, the gap between efficacious interventions and their application in the real-life setting, can be bridged through implementation research (IR) [10]. A scoping systematic review of implementation strategies in ECS identified 197 studies from 2000 to 2017 [11]. However, only 42% of these studies focused on identifying evidence-practice gaps, whereas 39% of these studies provided limited detail on the IR strategies. These include studies on trauma [12–17], sepsis [18–20] and cardiac emergencies [21, 22]. Few IR studies have focused on ECS issues such as triage in ED [18, 23]. But there is lack of studies focusing on comprehensive ECS delivery (ranging from triage to referral) [7]. The AIIMS-NITI study has provided concrete evidence of a fragmented ECS in India, covering all phases from prehospital to in-hospital and follow-up care. In the current scenario, trauma patients are sent to a designated trauma centre, while maternity and paediatric cases are channelled to specialized facilities for mothers and children [24]. This situation highlights the critical need for an integrated system that can provide emergency treatment to any patient with a time-sensitive condition within a single, cohesive emergency care structure. Hence, the authors aim to conduct this novel IR study on integrated ECS delivery to address these gaps and enhance patient care.

## Objectives

The objectives of this study are (a) to identify the barriers and facilitators of emergency care delivery for patients with time-sensitive conditions and (b) to develop and implement a contextually relevant model, measuring its impact through implementation research outcomes such as acceptability, adoption, fidelity and penetration.

## Methods

### Study settings

**Fig. 1:**
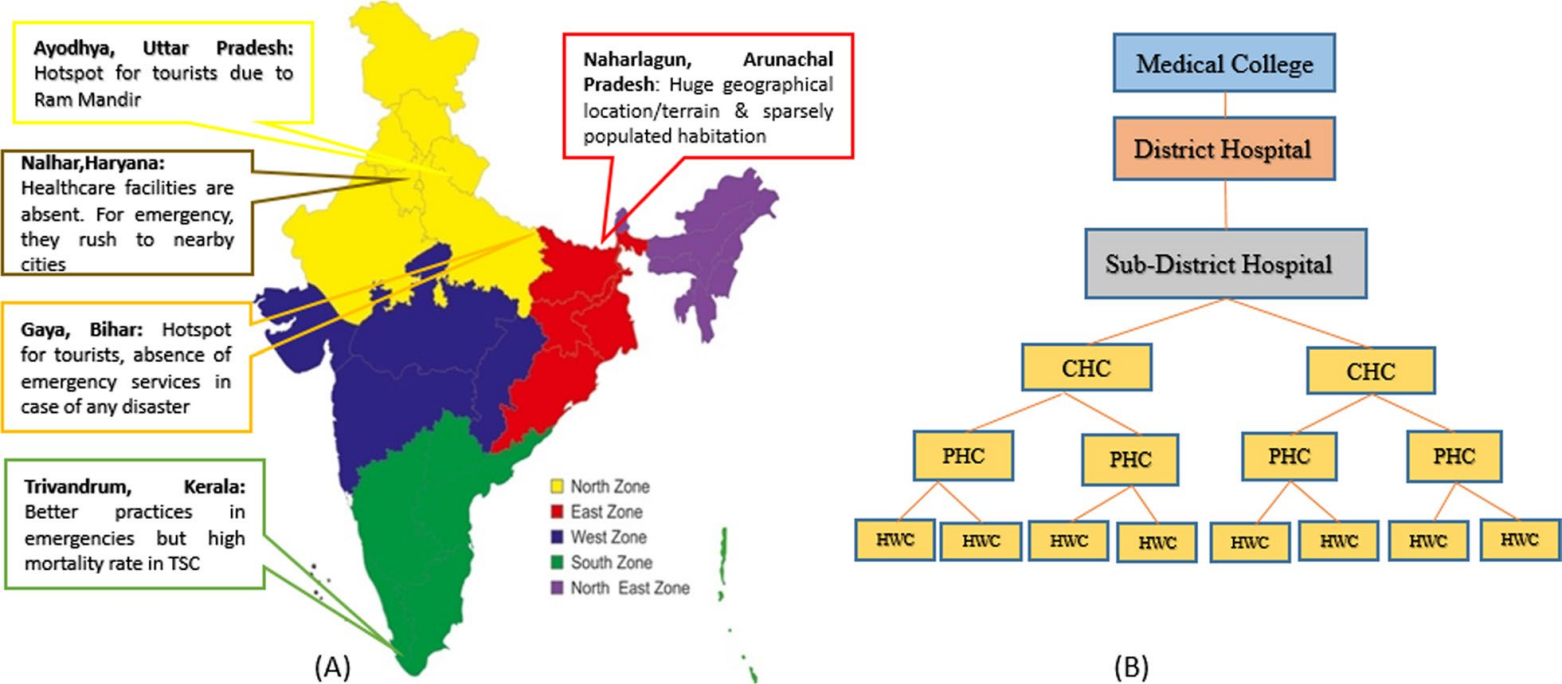
**a** Map of India depicting five zones, **b** presentation of a chain of linked healthcare facilities. *CHC* community health centre, *PHC* primary health centre, *HWC* health and wellness centre

This multi-centric study will be carried out in healthcare facilities (HCF) of five zones of India, that is, the north-west, north, east, south and north-east regions (Fig. 1a further lists the different study sites along with the inclusion criteria). The study period is 3 years and will be centrally coordinated from All India Institute of Medical Sciences (AIIMS) and ICMR, New Delhi. In India, the public health system is divided into the primary level (primary health centre and sub-centre), secondary level (sub-district hospital and community health centre) and tertiary level (medical college and district hospital) [25]. For the study, one chain of HCF (from primary level to tertiary level of care) will be selected from each zone. Each chain will consist of a total of 17 HCFs, that is, one medical college, one district hospital (DH), one sub-district hospital (SDH), two community health centres (CHC), four primary health centres (PHC) and eight sub-centres/health and wellness centres (HWC; Fig. 1b). So, a total of 85 HCFs were selected.

These five study zones were selected for this study through purposive sampling, with a total of 17 healthcare facilities (HCF) or sites chosen in each zone. Naharlagun in Arunachal Pradesh was chosen due to its challenging geographical terrain and sparsely populated areas, which make providing healthcare facilities difficult. Ayodhya in Uttar Pradesh was included because it is a significant site for mass gatherings due to religious tourism. Nalhar in Haryana was selected because of its sparse healthcare facilities, causing residents to seek emergency services in nearby cities. Gaya in Bihar is a tourist hotspot that needs enhanced emergency services for potential disasters. Trivandrum in Kerala was chosen for comparison due to its relatively better healthcare practices, allowing us to adapt and implement best practices. These zones have been purposefully chosen because, according to the Global Burden of Diseases – 2021 report, the majority of deaths in these regions were related to ischemic heart diseases, stroke, breathlessness, trauma and infections [26].

### Team description

The study team will comprise four groups: the central coordination team, the technical support unit, the implementers and the research teams. The detailed roles and responsibilities are as follows:

1. Central Coordination Team (at AIIMS New Delhi): This team will be responsible for the overall study management, coordination and oversight. It will consist of emergency medicine consultants and project scientists who will coordinate with project site staff to ensure the smooth implementation of the study protocol.
2. Technical support unit (at each study zones): The technical support unit (TSU) will consist of key administrative stakeholders from the state, medical colleges, districts and healthcare facilities (HCF). These stakeholders include state and district health authorities (Ministry of Health and Family Welfare officials, the Health Secretary, the director of Health Services, State National Health Mission officials and chief district medical Officers), as well as facility administrators (principals, medical superintendents, chief medical officers and chief nursing officers). Subject experts from emergency medicine, public health and external support organizations with expertise in implementation research are also part of this TSU. Their primary role will be to oversee local implementation efforts and ensure alignment with the directives from the central coordination team. They will participate in stakeholder meetings to assess the barriers and facilitators of ECS and will plan the individual implementation strategies. Additionally, they will act as liaisons between the central coordination team and local arrangements, facilitating effective communication and collaboration.
3. Implementers (at each facility): The implementers’ team will consist of key individuals from the facility administration, and facility stakeholders, including ED physicians, ED nurses, supporting healthcare staff and ambulance personnel. The ED team members will be responsible for providing care in terms of recognition, resuscitation and referral (3R) for TSC. They will be responsible for implementing the decided strategies at the facilities. They will follow the guidance of TSU and CCT, and provide feedback during meetings with the facility administrators.
4. Research teams (at each facility): The research team members will be recruited through this IR project and will be deployed at each facility during the study period. They will be retracted once a sustainable model is developed and implemented successfully. This team includes multiple sub-teams with specific roles:
  o Formative research and program evaluation team: Comprising project scientists, this team will conduct formative research and concurrent evaluations every 3 months to improve the implementation strategies. They will analyse collected data for learning and feedback. Project scientists in this team will conduct baseline, midline and end-line assessments, collecting both qualitative and quantitative data. This sub-team will provide feedback to the TSU and implementers.
  o Implementation support team (IST): This team will consist of nurses (emergency nurse coordinators) who will be available at the site throughout the implementation. They will assist implementers in applying the implementation strategies, help in clinical work, do data collection and perform community engagement.

### Implementation phases

**Fig. 2:**
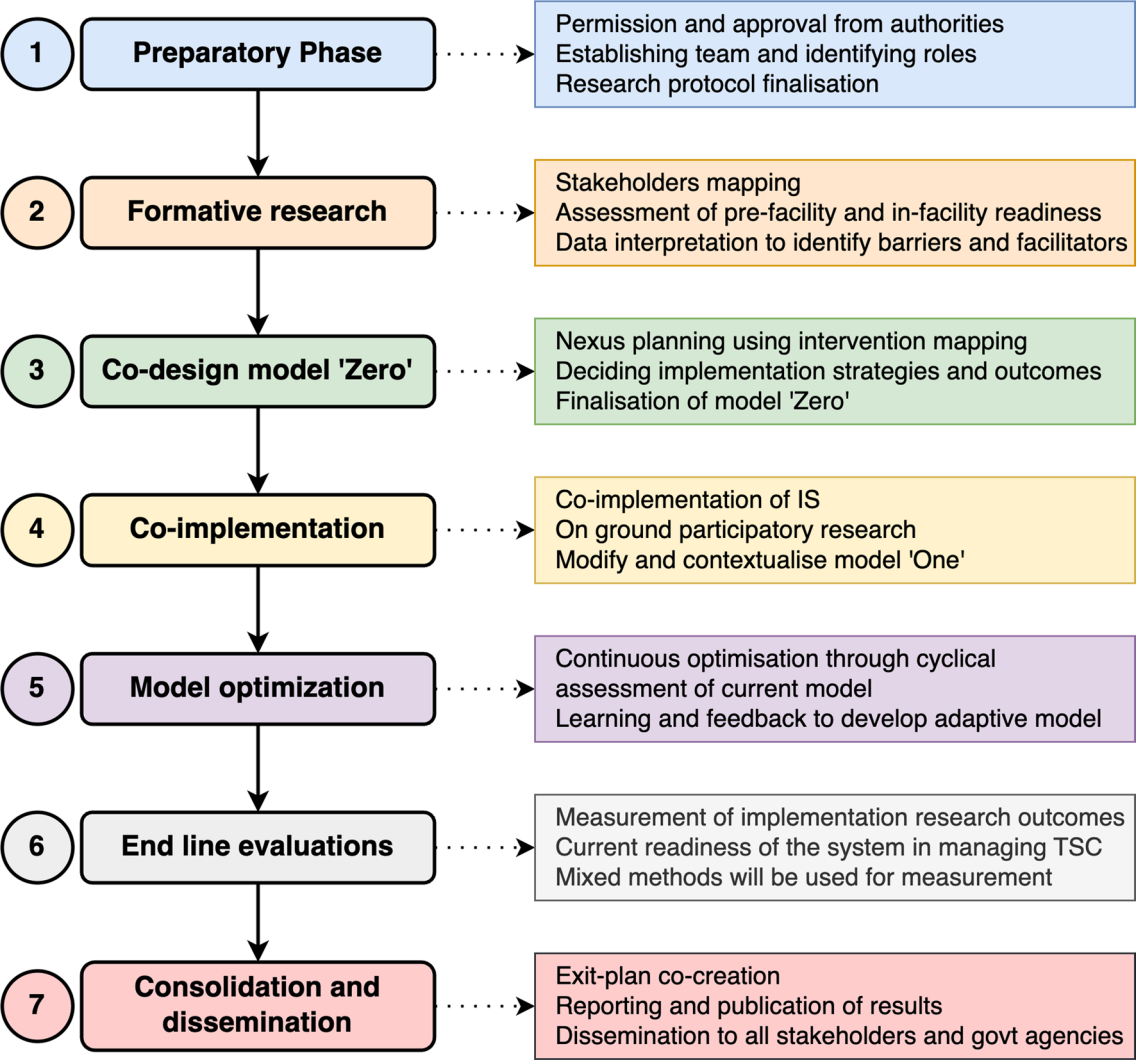
Phases of implementation planned in this study. *IS* implementation strategies, *TSC* time-sensitive conditions

The study will be conducted in seven phases, that is, the preparatory phase, formative research, co-design of Model “Zero”, co-implementation, modification of Model “Zero”, end-line evaluations, and consolidation and dissemination of results. This implementation strategy is summarized in Fig. 2.

### Phase 1 – Preparatory phase

The research protocol for the study sites was finalized with due permission and approval from the authorities. The next step is to establish a team and identify the roles of different team members at the central coordinating centre along with the team allocated to different sites. Further selection of chain of hospitals from different levels of healthcare facilities such as primary, secondary and tertiary was done in the respective districts.

### Phase 2 – Formative assessment

In Phase 2, we will conduct formative research using a mixed methods approach, which includes both quantitative and qualitative assessments. The research will consist of several components: stakeholder mapping; assessment of the community, prehospital system and emergency department readiness for managing TSC; and data interpretation to identify and tailor implementation strategies for the following phase. Stakeholder mapping will be conducted through in-depth interviews and focus group discussions with key stakeholders, including state and district health authorities, facility administrators, healthcare providers, patients and ambulance personnel. These discussions will help us identify the necessary stakeholders to discuss barriers, facilitators of ECS for TSC and implementation strategies.

The readiness of the community will be assessed through interviews with the general public and bystanders/relatives of the patients arriving at the healthcare facilities. The readiness of the prehospital infrastructure will be evaluated using quantitative tools, live observation of ambulance system processes and qualitative interviews with ambulance personnel, command centre staff and their administrators. The readiness of the emergency department regarding the management of TSC will be assessed using quantitative tools such as surveys, questionnaires, existing performance data, clinical records and process documentation. Additionally, the research team will conduct live observations of the ED processes and carry out interviews, including in-depth interviews with facility administrators, physicians and nurses, as well as focus group discussions with allied healthcare staff.

The findings from this formative research will inform the subsequent phases of the study. Specifically, the data will be used to refine and tailor interventions on components of 3R in each TSC, to address site-specific barriers and facilitators and to develop targeted strategies for improving implementation processes. This approach ensures that the research is context-specific and directly applicable to the study sites and also the best practices from other sites are found, providing a robust foundation for refining and improving our implementation strategies.

### Phase 3 – Co-design of Model “Zero”

The analysis of the formative research data will help us identify gaps, facilitators, barriers, good practices and opportunities. Following this analysis, nexus planning will be conducted in collaboration with the CCT, TSU and local stakeholders from each district. Nexus planning involves organizing meetings with relevant stakeholders, including chief investigators from CCT, subject experts, site investigators, key administrative stakeholders from the state, medical colleges, districts, healthcare facilities (HCF), facility administration and implementers such as senior ED physicians and senior ED nurses. The first implementation model, known as Model “Zero”, will be developed on the basis of the insights gained from these meetings. The development of Model “Zero” will follow the intervention mapping framework as outlined by Powell et al., which consists of five steps: needs assessment, defining proximal program objectives, enlisting intervention methods, designing implementation strategies, and monitoring and evaluation [27]. The needs assessment has already been completed during the formative research phase. We will then specify the objectives of the implementation model, focusing on recognition, resuscitation and referral (3R) of time-sensitive conditions. Intervention methods will be generated to align with these objectives, tailored to address the specific barriers and facilitators identified in the formative research. These methods will be operationalized into detailed implementation strategies, outlined in Table 1 and Supplementary Table 1, which will guide the activities for each of the 3R components. Finally, the research team will monitor the implementation process and evaluate the research outcomes (Table 2; Supplementary Table S2).

**Table 1:** Description of 3R at different levels of facility and tailored implementation strategies planned

3R components	Pre-facility level	In-facility level
Recognition	Development of effective educational materials and dissemination in the community	Setting up triage data collection system
	Community-based training (recognition of TSC and using the ambulance system)	Creation of triage teams and protocol in the emergency department
		Standard operating protocol for triage
		Training of the trainers (senior physicians and nurses)
		Train triage personnel in national triage protocol
		Frequent triage audits
		Infrastructure development and maintenance (setting up triage desk, monitors, etc.)
Resuscitation	Development of effective educational materials and dissemination in the community	Development of clinical pathways and standard operating protocols for TSC
	Community-based training on TSC	Development/reorganization of ED resuscitation bay
	Infrastructure and equipment availability in the ambulances	Development of leadership involvement
	Training of ambulance personnel regarding prehospital resuscitation	Training of the trainers
	Development of prehospital data collection system	Conducting of ongoing training of ED staff
	Standard operating protocol implementation	Development of academic partnership
		Conducting of quality improvement projects
		Development of quality monitoring systems (frequent audits)
		Identification of local champions of change
Referral		Development of referral record system
		Standard operating protocol for referral
		Monthly audits of referrals with administrators
		Information sharing between healthcare facilities regarding capabilities of handling TSC
		Continuous training of ED healthcare staffs on referral policy

*3R* recognition, resuscitation and referral, *TSC* time-sensitive conditions, *ED* emergency department

**Table 2:** Implementation research outcomes planned according to the implementation strategies

Implementation research outcome	Detailed implementation research outcomes	Assessing which implementation strategy
Acceptability	Acceptability of community training among general public	Development of effective educational materials and dissemination, and training for it in the community
	Acceptability and adoption of prehospital training among ambulance personnel	Training of ambulance personnel regarding prehospital resuscitation
	Acceptability of triage implementation among the triage staff	Training of triage personnel in national triage protocol
	Acceptability of SOP of TSC implementation among triage staff	Conducting of ongoing training of ED staff
	Acceptability of SOP for referral of patients with TSC	Continuous training of ED healthcare staff on referral policy
Adoption	Adoption of triage by triage teams	Train triage personnel in national triage protocol
	Adoption of SOP for management of TSC	Conducting of ongoing training of ED staff
	Adoption of referral policy	Continuous training of ED healthcare staff on referral policy
Fidelity	Fidelity of triage implementation: adherence to the triage guidelines as prescribed	Training of triage personnel in national triage protocol
	Fidelity of resuscitation of each TSC: adherence to the resuscitation guidelines	Developing clinical pathways and standard operating protocols for TSC
	Fidelity of referrals of each TSC	Continuous training of ED healthcare staffs on referral policy
Penetration	Penetration of adequate infrastructure and equipment availability in the ambulances	Infrastructure and equipment availability in the ambulances
	Penetration of prehospital data collection system	Development of prehospital data collection system
	Penetration of triage implementation (designated triage register) at the facility level	Setting up of triage data collection system
	Penetration of triage implementation (designated triage team) at the facility level	Creation of triage teams and protocol in the emergency department
	Penetration of adequate infrastructure for triage	Infrastructure development and maintenance (setting up triage desk, monitors, etc.)
	Penetration of proper ED resuscitation bay at the facility level	Development/reorganization of ED resuscitation bay
	Penetration of referral record system at the facility level	Development of referral record system

*SOP* standard operating procedures, *TSC* time-sensitive conditions, *ED* emergency department

The planned implementation strategies with respect to 3R components are provided in Table 1 and relevant details are available in Supplementary Table S1. Regarding recognition, our strategies at the pre-facility level will involve creating and distributing educational resources within the community, providing training on identifying TSC and making use of the ambulance system. At the in-facility level, the focus will be on the implementation of a data collection system for triage, the formation of triage teams, the establishment of standardized protocols, the training of personnel, the regular auditing of processes and the development of essential infrastructure. In terms of resuscitation, pre-facility efforts involve similar educational and training initiatives, focusing on prehospital resuscitation, ensuring the availability of infrastructure and equipment and development of a prehospital data collection system. At the in-facility level, the focus shifts to developing clinical pathways, standard operating protocols, the reorganization of ED resuscitation bays, trainer training, continuous staff training, academic partnerships, quality improvement projects and identification of local champions for change. Finally, for improving the referral system, the initial methods will involve developing referral protocols and systems before patients are admitted to a HCF. For patients in the facility, efforts are made to standardize referral protocols, conduct monthly audits, promote information exchange and train healthcare staff on referral policies.

To evaluate successful implementation, four implantation research outcomes (IRO) will be investigated, that is, “Acceptability”, “Adoption”, “Fidelity” and “Penetration”, as proposed by Proctor et al. [28]. For each implementation strategy (described in Table 1), we will evaluate the implementation process using these IROs. The detailed implementation research outcomes and their corresponding implementation strategies are presented in Table 2, and proposed calculations are provided in Supplementary Table S2. We will evaluate the acceptability of community training among the general public, prehospital training among ambulance personnel, triage implementation among triage staff, SOP for TSC management among triage staff and SOP for referral of patients with TSC. The adoption of an implementation strategy will be assessed in the initial phase of the study. We will examine the adoption of triage by triage teams, SOP for TSC management and referral policy. We will investigate the fidelity of triage implementation in adherence to prescribed guidelines, resuscitation adherence for each TSC and fidelity of referrals. We will assess the penetration of our implementation strategy in the end-line evaluation phase. Penetration of infrastructure readiness and equipment availability in ambulances, prehospital data collection systems, triage implementation at the facility level, triage team implementation at the facility level, infrastructure for triage, proper ED resuscitation bays as per standards and referral systems at the facility level. These assessments will provide a comprehensive evaluation of the implementation process, ensuring that each strategy is effectively contributing to the desired outcomes.

### Phase 4 – Co-implementation

After the nexus planning for developing Model “Zero”, which focuses on recognition, resuscitation and referral (3R); TSC; and site-specific strategies, the implementation of the planned strategies will be carried out collaboratively by the site implementers, the research team and the implementation support team of the IR project. This phase will involve the execution of all relevant planned implementation strategies as detailed in Table 1 and Supplementary Table S1. These strategies encompass various aspects, including targeted skill enhancement training, process improvements, resource allocation and quality control measures. The on-ground participatory research will facilitate the modification and contextualization of Model “One” and subsequent models, ensuring that the strategies remain tailored and effective in the local context.

### Phase 5 – Model optimization

**Fig. 3:**
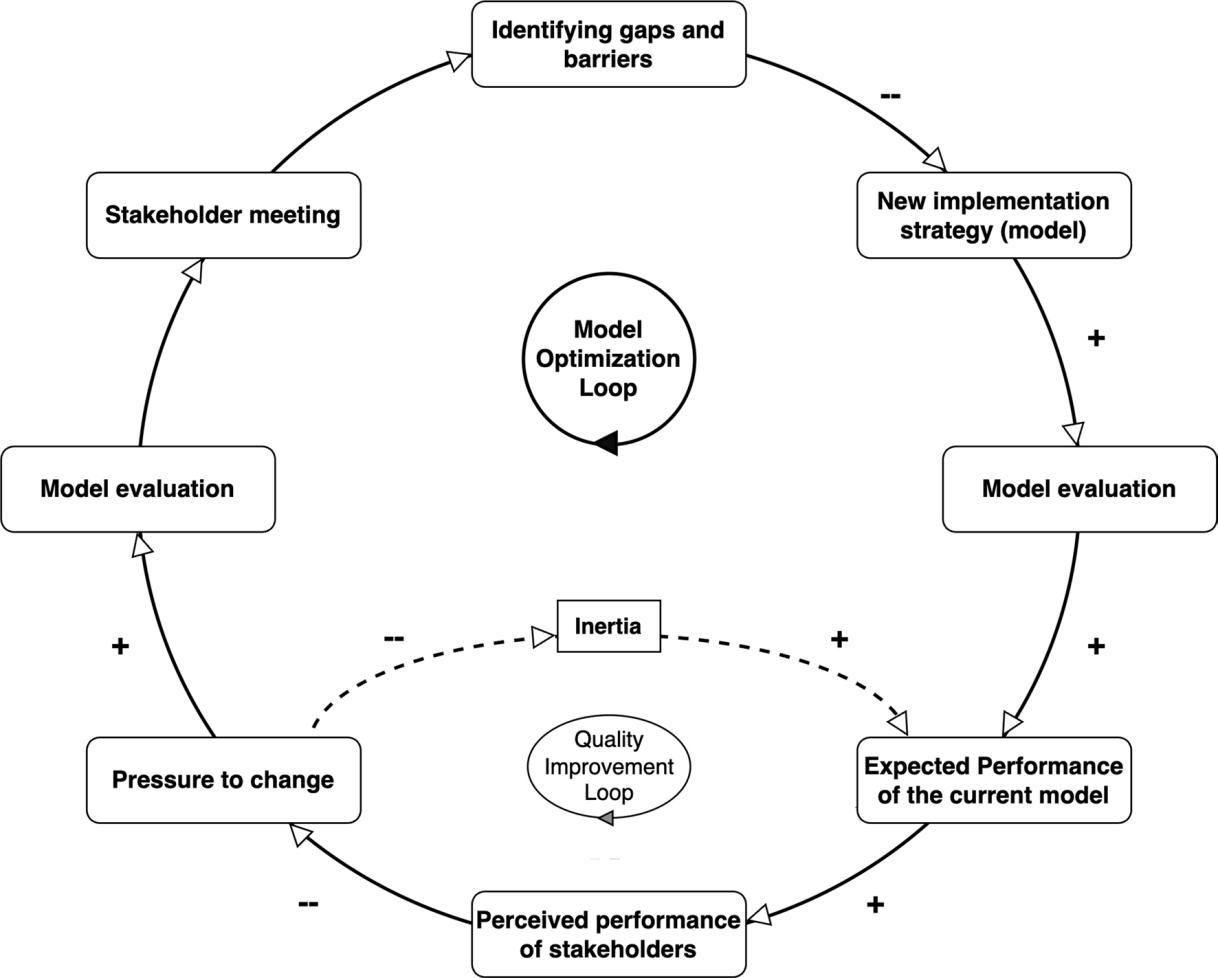
Model optimization loop for implementation strategy improvement. This figure illustrates the continuous process of identifying gaps and barriers, implementing new strategies, evaluating the model and considering stakeholder perceptions. The ± signs indicate the positive or negative influence of each step on the process. Positive signs (+) indicate steps that enhance the process, while negative signs (–) indicate steps that may introduce challenges or resistance (inertia). The loops emphasize ongoing evaluation and adjustment to improve the model’s performance and address stakeholder feedback. Quality improvement loop is a component of the larger model optimization loop

In Phase 5, we focus on the continuous optimization of the implementation model as shown in Fig. 3. On the basis of the insights from formative assessment (phase 2), new implementation strategies will be developed to address the identified issues (phase 3). These strategies will then be co-implemented (phase 4). The “Model optimization” phase will be a cyclical phase consisting of phases 2–4; evaluation of the performance of the current model, which will be closely monitored; assessment of model’s effectiveness using IROs; and interviewing stakeholders for perceived performance to gauge its success [27]. If the performance does not meet the desired standards, internal quality improvement initiatives will be undertaken as per Deming’s cycle of Plan-Do-Study-Act [29]. These initiatives aim to enhance performance and address any residual inertia within the system. The entire process is cyclic, involving regular model evaluations and stakeholder meetings to ensure that the implementation strategies remain effective and responsive to the evolving needs of the study sites. This iterative loop of assessment, implementation and evaluation ensures that the model is continually refined and optimized for better outcomes.

### Phase 6 – End-line evaluations

The end-line evaluation will measure implementation-related outcomes (IRO) such as acceptability, adoption, fidelity and penetration as outlined in Table 2 and Supplementary Table S2. The readiness of the community, prehospital services and emergency departments (ED) for managing TSC will be reassessed similarly to the formative assessment. Quantitative tools such as surveys, performance data, clinical records and live observations of ED processes, along with qualitative methods such as in-depth interviews with facility administrators, physicians and nurses, and focus group discussions with allied healthcare staff, will be used to evaluate the final model. This final model will be tailored to the specific context (geographic location and level of HCF).

### Phase 7 – Consolidation and dissemination of results

The study team will withdraw the support system at HCFs as an exit plan. The research team will conduct a meeting with the ISU to prepare an effective implementation plan based on the lessons from the comprehensive through end-line evaluations. The report writing will be done after a detailed data analysis of all the gathered data. The results of the IR study will be disseminated to all stakeholders (local, district, state and national level).

### Data collection and analysis

Mixed-methods research will be conducted in this implementation research project. For the qualitative study, researchers at specific sites will conduct audio recordings with the consent of the interviewees. The recorded interviews will be transcribed verbatim and translated into English. To ensure accuracy, 10% of the transcriptions will be validated by re-listening to the recordings. Thematic analysis with inductive coding will be performed to understand the context and identify barriers and facilitators of emergency services for TSC at pre-facility and in-facility levels [30]. This analysis will be done according to interviewee category (physicians, nurses, administrators, etc.), site and healthcare facility (HCF) level. We will compare the zone-wise barriers and facilitators and create frequency tables for each domain and subdomain to list the barriers and facilitators on the basis of the constructs.

For the quantitative methodology, the readiness of the system will be assessed using quantitative tools and live observations of TSC management at each HCF across different zones as described in the “Phase 2 – Formative assessment”. Quantitative variables will be represented as means or medians, while categorical variables will be represented as frequencies and percentages. From the combined quantitative and qualitative data, IRO such as “Acceptability”, “Adoption”, “Fidelity” and “Penetration” will be derived. Detailed calculations for IRO are provided in Supplementary Table 2. “Acceptability” and “Adoption” will be assessed in the early phase of the study, while “Fidelity” and “Penetration” of intervention strategies will be evaluated at 3-month intervals to monitor trends and inform “Phase 5 – Model optimization”. Quantitative data analysis will be performed using the latest version of SPSS, and qualitative data analysis will be conducted using the latest version of NVivo.

## Discussion

This study aims to address critical gaps in India’s emergency care systems by implementing an integrated model focusing on time-sensitive conditions. We will use a multi-phase approach, including formative assessment, model co-design and co-implementation to ensure strategies are contextually relevant and tailored to the specific needs of each healthcare facility. The combination of qualitative and quantitative methodologies will provide a comprehensive understanding of barriers and facilitators to effective ECS delivery, and the iterative model optimization will ensure continuous improvement.

During the formative assessment phase, we will identify key gaps in community readiness, prehospital infrastructure and emergency department management of TSC. These insights will inform the development of Model “Zero” through collaborative nexus planning with key stakeholders. In the co-implementation phase, we will execute targeted strategies such as skill enhancement training, process improvements and resource allocation, tailored to each facility’s needs. This participatory approach will facilitate model adaptation to local contexts, ensuring its effectiveness and sustainability. In the model optimization phase, we will refine strategies through continuous evaluation and stakeholder feedback. The end-line evaluation will assess the impact of these strategies on implementation-related outcomes, such as acceptability, adoption, fidelity and penetration, using quantitative and qualitative tools. This comprehensive evaluation framework will ensure that the final model is both effective and adaptable to various healthcare settings. At the end, we will implement an exit strategy, gradually withdrawing research support from healthcare facilities while finalizing an effective implementation plan. The findings will be disseminated to stakeholders at all levels to ensure sustainable adoption and adaptation of the integrated emergency care system model.

## Conclusion

This study will systematically conduct implementation research on India’s emergency care systems by focusing on time-sensitive conditions through a contextually tailored, integrated model. The multi-phase implementation strategy, incorporating both qualitative and quantitative methodologies, will ensure continuous optimization and effectiveness. The findings from this research will provide valuable insights into the barriers and facilitators of ECS delivery and contribute to developing sustainable, high-quality emergency care systems in low- and middle-income countries.

## Supplementary Information

Additional file 1.

## Data Availability

No datasets were generated or analysed during the current study.

## Abbreviations

AIIMS: All India Institute of Medical Sciences
CCT: Central coordinating team
ECS: Emergency care system
ED: Emergency department
FGD: Focused group discussion
GBD: Global burden of disease
HCF: Healthcare facilities
ICMR: Indian Council of Medical Research
IDI: In-depth interview
IR: Implementation research
IST: Implementation support team
LMIC: Low- and middle-income countries
NFI: Non-informal interactions
NITI: National Institute of Transforming India
TSU: Technical support unit
