# Supplementary material for: Strengthening facility-based integrated emergency care services for time sensitive emergencies at all levels of healthcare in India: An implementation research study protocol
Source: Health Res Policy Syst. 2024 Sep 9;22:125. doi: 10.1186/s12961-024-01183-x (PMC11382461; doi:10.1186/s12961-024-01183-x)
Supplement: Supplementary file 1 — Additional file 1. [file 12961_2024_1183_MOESM1_ESM.docx]

**Supplementary Appendix**

**Supplementary Table – 1:** Description about 3R at different levels of facility and detailed implementation strategy

**Supplementary Table – 2:** Details of Implementation research outcomes planned according to the implementation strategies

**Supplementary Table – 1: Description about 3R at different levels of facility and detailed implementation strategy**

| **Facility** | **3R components** | **Probable implementation strategies** | **Actor** | **Target** |
| --- | --- | --- | --- | --- |
| **Pre-facility** | **Recognition** | Development of effective educational materials and dissemination in the community | Designated healthcare providers at healthcare facilities | Community |
|  |  | Community-based training (recognition of TSC and using ambulance system) | Designated healthcare providers at healthcare facilities | Community |
|  | **Resuscitation** | Development of effective educational materials and dissemination in the community | Designated healthcare providers at healthcare facilities | Community |
|  |  | Community-based training on TSC | Designated healthcare providers at healthcare facilities | Community |
|  |  | Infrastructure and equipment availability in the ambulances | Administrators of ambulance services in the locality. | Ambulances |
|  |  | Training of ambulance personnel regarding prehospital resuscitation | Designated healthcare providers at healthcare facilities | Ambulance personnel |
|  |  | Development of prehospital data collection system | Administrators of ambulance services in the locality and IT support from government | Prehospital data collection system |
|  |  | Standard operating protocol implementation | Designated healthcare providers at healthcare facilities, state and district health authorities, and field experts | Ambulance and ambulance personnel |
| **In-facility** | **Recognition** | Setting up triage data collection system | Administrators / in charge of the department / HCF, IT staffs of the HCF or state government, and subject experts from local medical college and AIIMS | Triage data collection system |
|  |  | Create triage teams and protocol in the emergency department | Administrators / in charge of the department / HCF, subject experts from local medical college and AIIMS | Triage team formation |
|  |  | Standard operating protocol for triage | Administrators / in charge of the department / HCF, subject experts from local medical college and AIIMS | Standard operating protocol for triage |
|  |  | Training of the trainers (senior physicians and nurses) | Subject experts from the AIIMS | Senior physicians and nurses |
|  |  | Train triage personnel in national triage protocol | Senior physicians and nurses | Designated staffs of the triage team (physicians, nurses, and/or paramedical staffs) |
|  |  | Frequent triage audits | Administrators / in charge of the department / HCF | Triage audits |
|  |  | Infrastructure development and maintenance (setting up triage desk, monitors, etc) | Administrators / in charge of the department / HCF | Triage area |
|  | **Resuscitation** | Developing clinical pathways and standard operating protocols for TSC | Administrators / in charge of the department / HCF, senior physicians and nurses of ED, and subject experts from local medical college and AIIMS | Clinical pathways and standard operating protocols for management of TSC |
|  |  | Development / Reorganisation of ED resuscitation bay | Administrators / in charge of the department / HCF, senior physicians and nurses of the ED | Resuscitation bay |
|  |  | Develop leadership involvement | Administrators / in charge of the department / HCF, State / district health authorities | Leadership involvement |
|  |  | Training of the trainers | Subject experts from the AIIMS | Senior physicians and nurses |
|  |  | Conduct ongoing training of ED staffs | Senior physicians and nurses | Designated staffs of the ED resuscitation team (physicians, nurses, and/or paramedical staffs) |
|  |  | Develop academic partnership | Senior physician and nurses of medical college with subject experts from AIIMS | Academic partnership |
|  |  | Conducting quality improvement projects | Senior nurses and physicians | ED quality improvement projects |
|  |  | Develop quality monitoring systems (frequent audits) | Administrators / in charge of the department / HCF, senior physicians and nurses of the ED | ED quality monitoring related to TSC |
|  |  | Identifying local champions of change | Administrators / in charge of the department / HCF, and AIIMS experts | Designated staffs of the ED resuscitation team |
|  | **Referral** | Development of referral record system | Administrators / in charge of the department / HCF | Referral record system development (correct documentation of referrals for patients and for the HCF) |
|  |  | Standard operating protocol for referral | Administrators / in charge of the department / HCF, subject experts from local medical college and AIIMS | Standard operating protocol for referral |
|  |  | Monthly audits of referrals with administrators | Administrators / in charge of the department / HCF | ED quality monitoring related to referrals |
|  |  | Information sharing between healthcare facilities regarding capabilities of handling TSC | Administrators / in charge of the department / HCF, IT team, and state / district health authorities | Common information sharing between nearby HCF |
|  |  | Continuous training of ED healthcare staffs on referral policy | Senior physicians and nurses | Staffs of ED |

**Supplementary Table – 2: Details of Implementation research outcomes planned according to the implementation strategies**

| **3R components** | **Probable implementation strategies** | **Implementation research outcomes** | **Operational definition of IRO** |
| --- | --- | --- | --- |
| **Recognition (Pre-facility)** | Development of effective educational materials and dissemination in the community | Acceptability of interventions will be assessed by conducting pre- and post-training interviews of the general public. | % Acceptability (n = person who positively accepts the training information, d = person who are provided with the educational materials and/or training) |
|  | Community-based training (recognition of TSC and using ambulance system) | Acceptability of interventions will be assessed by conducting pre- and post-training interviews of the general public. | % Acceptability (n = person who positively accepts the training information, d = person who are provided with the educational materials and/or training) |
| **Resuscitation (Pre-facility)** | Development of effective educational materials and dissemination in the community | Acceptability of interventions will be assessed by conducting pre- and post-training interviews of the general public. | % Acceptability (n = person who positively accepts the training information, d = person who are provided with the educational materials and/or training) |
|  | Community-based training on TSC | Acceptability of interventions will be assessed by conducting pre- and post-training interviews of the general public. | % Acceptability (n = person who positively accepts the training information, d = person who are provided with the educational materials and/or training) |
|  | Infrastructure and equipment availability in the ambulances | Penetration of adequate infrastructure and equipment availability in the ambulances | % Penetration (n = number of ambulances with adequate infrastructure, d = number of ambulances covered) |
|  | Training of ambulance personnel regarding prehospital resuscitation | Acceptability and Adoption of prehospital training among ambulance personnel | % Acceptability (n = person who positively accepts the training, d = person who are provided with the training), % Adoption (n = number of persons who has uptaken the training and managing TSC accordingly, d = number of ambulance personnel trained) |
|  | Development of prehospital data collection system | Penetration of prehospital data collection system | % Penetration (n = number of ambulances who have incorporated prehospital data collection, d = number of ambulances covered in IR) |
|  | Standard operating protocol implementation |  |  |
| **Recognition (In-facility)** | Setting up triage data collection system | Penetration of triage implementation (designated triage register) at the facility level. |  |
|  | Create triage teams and protocol in the emergency department | Penetration of triage implementation (designated triage team) at the facility level. | % Penetration (n = number of HCF which have designated a triage team, d = number of HCF in this project) |
|  | Standard operating protocol for triage |  |  |
|  | Training of the trainers (senior physicians and nurses) |  |  |
|  | Train triage personnel in national triage protocol | Acceptability of triage implementation will be assessed by conducting pre- and post-training interviews of the triage staffs and senior stakeholders of the department specifically in terms of intuitive appeal and likelihood of application in the duty, Adoption of triage by triage teams, Fidelity of triage implementation: Adherence to the triage guidelines as prescribed. | % Acceptability (n = person who positively accepts the training information, d = person who are provided with the educational materials and/or training). % Adoption of triage (n = physician or nurses who have adopted the triage, d = total number of physician or nurses trained), % Fidelity (n = number of triage staffs correctly doing triage as prescribed in the SOP, d = number of triage staffs trained on triage) |
|  | Frequent triage audits |  |  |
|  | Infrastructure development and maintenance (setting up triage desk, monitors, etc) | Penetration of adequate infrastructure for triage | % Penetration (n = number of HCF with adequate infrastructure at triage, d = number of HCF in this study) |
| **Resuscitation (In-facility)** | Developing clinical pathways and standard operating protocols for TSC | Fidelity of resuscitation of each TSC | % Fidelity (n = number of physicians correctly resuscitating patients with a TSC as prescribed in the SOP, d = number of physicians trained) |
|  | Development / Reorganisation of ED resuscitation bay | Penetration of proper ED resuscitation bay at the facility level, as per the standards mentioned in National Accreditation Board for Hospitals and Healthcare providers | % Penetration (n = number of HCF which have a proper resuscitation bay, d = number of HCF in this project) |
|  | Develop leadership involvement |  |  |
|  | Training of the trainers |  |  |
|  | Conduct ongoing training of ED staffs | Acceptability of SOP of TSC implementation will be assessed by conducting pre- and post-training interviews of the triage staffs and senior stakeholders of the department specifically in terms of intuitive appeal and likelihood of application in the duty, Adoption of TSC management SOP | % Acceptability (n = physicians / nurses who positively accepts the training information, d = physicians / nurses who are provided with the educational materials and/or training). % Adoption of triage (n = physician / nurses who have adopted the TSC management guidelines, d = total number of physician or nurses trained). |
|  | Develop academic partnership |  |  |
|  | Conducting quality improvement projects |  |  |
|  | Develop quality monitoring systems (frequent audits) |  |  |
|  | Identifying local champions of change |  |  |
| **Referral (In-facility)** | Development of referral record system | Penetration of referral record system at facility level | % Penetration (n = number of HCF with referral record system, d = number of HCF in this study) |
|  | Standard operating protocol for referral |  |  |
|  | Monthly audits of referrals with administrators |  |  |
|  | Information sharing between healthcare facilities regarding capabilities of handling TSC |  |  |
|  | Continuous training of ED healthcare staffs on referral policy | Acceptability of SOP for referral of patients with TSC, Adoption of referral policy, Fidelity of referrals of each TSC. | % Acceptability (n = physicians who positively accepts the referral policy, d = physicians who are provided training on referral policy). % Adoption of referral system (n = physicians who have adopted the referral policy in there practice, d = total number of physician trained), % Fidelity (n = number of physicians correctly referring patient as prescribed in the SOP, d = number of physicians trained) |
